# Hydroxychloroquine Attenuates Acute Inflammation (LPS)-Induced Apoptosis via Inhibiting TRPV1 Channel/ROS Signaling Pathways in Human Monocytes

**DOI:** 10.3390/biology10100967

**Published:** 2021-09-27

**Authors:** Mustafa Güzel, Orhan Akpınar

**Affiliations:** 1 Labaratory of Medical Microbiology, Private Medical Center of Maltepe, Maltepe Tıp Merkezi, TR-34854 Istanbul, Turkey; 2 Medical Microbiology Unit, Oral and Maxillofacial Surgery Department, Dentistry School, Suleyman Demirel University, TR-32260 Isparta, Turkey; orhanakpnr@hotmail.com; 3 Department of Medical Microbiology, Health Sciences Institute, Suleyman Demirel University, TR-32260 Isparta, Turkey

**Keywords:** apoptosis, acute inflammation, cytokines, oxidative stress, TRPV1 channel

## Abstract

**Simple Summary:**

LPS is a well-known agent in cell line models, including U937 monocytes, for inducing acute inflammation (INF). It is not known whether antioxidant HCQ, through the inhibition of TRPV1 in U937, can decrease oxidative monocyte toxicity and cell death. We investigated the modulator action of HCQ treatment through the modulation of TRPV1 on the levels of mROS, INF, and apoptosis in an LPS-stimulated U937 monocyte model. Acute INF activates apoptotic, inflammatory, and oxidant action through acute INF-dependent excessive cROS, MDA, cytokine generation, and Ca^2+^ influx in U937 human monocyte cells. Furthermore, treatment with acute INF increases TRPV1 and apoptotic marker (CAS3, CAS9, Bax, and Bcl-2) concentrations via downregulation of glutathione level and glutathione peroxidase activity in U937 monocytes. The acute INF-caused U937 oxidative stress and cytotoxicity is diminished by the treatment of HCQ and TRPV1 inhibitor (CPZ). In summary, treatment with HCQ and CPZ induced anti-inflammatory, anti-apoptotic, and antioxidant action via the inhibition of cROS, cytokine generation, and caspase activation.

**Abstract:**

Acute inflammation (INF) and apoptosis are induced in monocytes by the generation of several factors, including the products of cytosolic oxygen free radicals (cROS) and the excessive influx of Ca^2+^ via the stimulation of TRPV1. These are main factors in the etiology of monocyte activation-induced inflammatory and neurodegenerative diseases. Importantly, the protective action of hydroxychloroquine (HCQ) treatment via the inhibition of TRPV1 on the levels of inflammatory factors, cROS, and apoptosis in acute INF (lipopolysaccharide, LPS)-exposed neuronal cells was recently reported. However, the relationships between acute INF via TRPV1 activation and HCQ in monocytes have not been fully clarified yet. The cell membrane of U937 human monocytes contains natural TRPV1. In the study plan, we used U937 cells in four main groups, namely control, HCQ (60 μM for 48 h), INF (1 μg/mL LPS for 16 h), and HCQ + INF. The current data indicate that LPS-induced acute INF caused the upregulation of excessive cytosolic Ca^2+^ accumulation via the stimulation of TRPV1 in the cells. The treatment of INF additionally upregulated the levels of apoptosis and cytokines (IL6, IL1β, and TNFα), due to upregulated cROS and lipid peroxidation levels as well as upregulated generation of caspase -3 (CAS3) and -9 (CAS9) but a decrease in glutathione and glutathione peroxidase. The expression levels of TRPV1, Bax, CAS3, and CAS9 were also upregulated by the treatment of LPS. However, treatment with HCQ and TRPV1 blocker (capsazepine) modulated the levels of cytokines, caspases, cROS, Ca^2+^ influx, and apoptosis through the modulation of TRPV1 in the U937 that were stimulated with LPS. In summary, the present data suggest TRPV1 activation through the acute INF (LPS)-induced inflammatory, oxidant, and apoptotic adverse actions in monocyte cells, whereas HCQ prevented adverse actions via the modulation of TRPV1. The results may be significant in the modulation of monocyte activation-caused inflammatory and neurodegenerative diseases.

## 1. Introduction

The host immune defense system in the blood is modulated by several types of immune cells such as lymphocytes, monocytes, and neutrophils [1]. The homeostasis of immune cells in the blood is arranged by several molecular pathways. The engulfed bacteria and viruses are killed by the immune cells via the release of pro-inflammatory mediators such as TNFα, IL1β, and IL6, and also the products of cytosolic oxygen free radicals (cROS) [2,3,4]. In the etiology of several inflammatory diseases such as lupus erythematosus (SLE), multiple sclerosis (MS), and psoriasis, [5,6], as well as neurodegenerative diseases [4,5], its stimulation is arranged by the Ca^2+^ influx [1,4]. In turn, simultaneous generation of cROS and cytokines via the excessive accumulation of the cytosolic free Ca^2+^ (cCa^2+^) into the mitochondria of the immune cells triggers adverse apoptosis and injury to tissues such as neurons, the skin, and the liver [4,5]. Hence, adverse oxidant and apoptotic action in the lymphocytes was decreased by treatment with Ca^2+^ influx modulator agents [7,8].

A key cell membrane component in Gram-negative bacteria is lipopolysaccharides (LPS), whose increase induces acute inflammation (INF) via the stimulation of leucocyte subgroups, including monocytes [4]. In nonimmune cells, the simulator action of LPS for initiating the processes of INF is also possible [9]. Hence, an increase in LPS in the plasma of patients with inflammatory and noninflammatory diseases was reported [9,10]. The inflammatory action of LPS causes adverse apoptotic and oxidant action via the excessive generation of mitochondrial ROS (mROS) and cROS in human monocytes [11]. Some chemicals induce antioxidant action, and the protective action of antioxidant chemicals against the adverse oxidant and apoptotic action of the acute INF in the monocytes was reported in several inflammatory diseases, including nonalcoholic fatty liver disease, psoriasis, and MS [11,12,13]. One of the antioxidant chemicals is chloroquine. A derivative of aminoquinoline is chloroquine, which is used in the treatment of malaria. Hydroxychloroquine (HCQ) also induces effective anti-inflammatory and antioxidant action in the treatment of diseases related to inflammation and oxidant stress such as rheumatoid arthritis and SLE [14,15]. In addition, chloroquine and HCQ induces antioxidant and anti-inflammatory action against LPS-induced adverse excessive INF and cytokine generation in the macrophages [15,16], although conflicting oxidant and inflammatory actions of chloroquine and HCQ are also present [17,18]. In addition to the anti-inflammatory actions of HCQ, its treatment downregulated the levels of pro-inflammatory cytokines, apoptosis, and mROS in several cell types [14,19,20,21,22]. Adverse oxidant and apoptotic actions are induced in inflammatory and neuronal cells by the excessive Ca^2+^ influx via the stimulation of the transient receptor potential (TRP) vanilloid 1 (TRPV1) channel [2,23,24,25]. The modulator role of HCQ in the TRPV1 cation channel in LPS-induced U937 monocytes was also recently reported [26].

A Ca^2+^-permeable cation channel superfamily in mammalian cells is TRP [27], which has 28 members, including TRPV1 [28]. TRPV1 is stimulated in U937 monocyte cells, the dorsal root ganglion (DRG), and the hippocampus by different stimuli, including the components of capsaicin (CAP) and oxidants such as hydrogen peroxide (H2O2) and nitric oxide (NO) [24,29]. Capsazepine (CPZ) acted as a blocker of TRPV1 in several cell types [2,24,29]. The LPS-mediated acute INF induces oxidative stress (OS) and stimulates TRPV1 in the DRG and lungs of mice [30,31]. LPS-mediated cytokine production and INF in the U937 were modulated by the blockage of TRPV1 [26]. The stimulation of TRPV1 causes an increase in the concentration of cCa^2+^, which accumulates in the mitochondria, resulting in an increase in the mitochondrial membrane potential [24]. In turn, it causes an increase in cROS, mROS, NO, and caspase levels [27]. In addition, the stimulation of TRPV1 via treatment with LPS induces apoptosis and mROS, although its stimulation was modulated by antioxidant treatments [9,10,32].

LPS is a well-known agent in monocyte line models for inducing acute INF [33,34]. It is not known whether antioxidant HCQ, through the inhibition of TRPV1 in U937 cells, can decrease oxidative monocyte toxicity and cell death. We investigated the modulator action of HCQ treatment through the modulation of TRPV1 on the levels of mROS, INF, and apoptosis in an LPS- stimulated U937 monocyte model.

## 2. Materials and Methods

### 2.1. Cell Culture

The U937 cells were gifted by Meltem Elitas (Sabancı University, Istanbul, Turkey). The natural expression of TRPV1 in the U937 monocytes was reported, and the cells were used in several studies of inflammation and TRPV1 activation [26,35,36]. Hence, the U937 cells in the current study were preferred for the experiment of TRPV1. The cells of U937 were kept in a medium of RPMI-1640 (Cat# RPMI-A and Capricorn), 10% fetal bovine serum (Cat #S0115, Sigma-Aldrich, Istanbul, Turkey), and 1% penicillin/streptomycin (Cat #15140148, Thermo Fisher, Istanbul, Turkey) [26,35]. After splitting the U937 cells into various T-25 flasks, they were kept in a NB-203QS automatic shaker incubator (Gyeonggi-do, South Korea).

### 2.2. The Experimental Groups

After counting the U937 cells with an CASY Modell TT automatic cell counter (Roche, Reutlingen, Germany), the cells were seeded in four T-25 flasks at an initial density of 1 × 10^6^ cells per mL, and divided into four groups: control (Cntr), HCQ, acute INF, and HCQ + INF. The Cntr cells were not treated with HCQ and LPS. After detecting a nontoxic dose (60 μM) of the HCQ, the cells in the group of HCQ were treated with 60 μM HCQ for 48 h. The cells of acute INF were treated with 1 μg/mL LPS (Cat #L2880-100MG, lipopolysaccharides from *Escherichia coli* O55:B5, Sigma-Aldrich) for 16 h [33]. In the HCQ + INF group, the cells were treated with LPS (1 μg/mL) for 16 h after treatment with HCQ (60 μM for 48 h). We induced subgroups in some analyses, and the cells in the groups were additionally treated with the CAP (10 μM) or CPZ (100 μM) for 30 min [23,24,25].

The stock solutions of CAP and CPZ were prepared in dimethyl sulfoxide (DMSO) under stirrer. Then, they were diluted to the appropriate concentration in the extracellular solution with Ca^2+^ (1.2 mM).

### 2.3. The Analyses of Cell Viability, Cell Count, Debris Amount, and Cell Volume

The cell viability, cell count, debris amount, and cell volume were assayed in the cell counter CASY TT (Roche) by using the CASY tone solution [37]. It is well known that viable cells have a full cell volume, which acts as a barrier to the current. However, the death of cells leads to a loss of cell volume and resistance in the cytosol. The analysis using the CASY counter was based on the presence or loss of resistance in the cytosol of the cells. The mean values of cell number, debris, and volume were indicated as 10^6^ cells/mL, although the cell viability values were expressed as percentage changes (%).

### 2.4. Western Blot Bands

The protein levels in the U937 cells were determined from the SDS-PAGE gels. The proteins were moved to the membranes of PVDF membranes. After diluting the primary antibodies (1:200), the obtained membranes were kept overnight at 4 °C with the diluted primary antibodies of anti-TRPV1 (Cat #PA-748; Thermo Fisher), CAS3 (Cat #14220; Cell Signaling Technology), CAS9 (Cat #9502; 1:200; Cell Signaling Technology), Bcl-2 (Cat #ab59348), Bax (Cat #ab53154) (Abcam), and β-actin (Cat #sc-47778) (Santa Cruz). After the incubation of secondary antibodies, the protein bands of the antibodies were visualized in the visible imaging system (G:Box, Syngene, Cambridge, UK). The band signal intensities of Bax, Bcl-2, CAS3, CAS9, β-actin, and TRPV1 were measured using ImageJ software (Version 1.48, NIH, USA) [37]. The internal control of the signal intensity was a housekeeping protein (β-actin antibody) band. The signal activities of bands were measured by using ImageJ software, and they were shown as relative density.

### 2.5. The Determination of Apoptosis, Cell Viability, CAS3, and CAS9

For the determination of apoptosis levels, we used a Biocolor APOPercentage apoptosis kit (Cat #A1000, County Antrim, Northern Ireland) [37,38]. For the apoptosis analyses, the cells were added to 24-black well plates, and the analyses were performed in an Infinite 200 PRO automatic plate reader (Tecan Ltd., Männedorf, Switzerland) after adding the required reagents.

In addition to the CASY analyses of cell viability, we performed a second analysis of cell viability (MTT). The MTT assay was applied to the cells as described in a previous study [38]. After diluting the cells in the CASY cell counter to a density of 10^6^ cells/well, the cells were added into 96-wellblack plates. After removing the RPMI medium from the cells, they were treated with 5 mg/mL MTT for 4 h at 37 °C. For dissolving the formazan crystals, DMSO treatment (100 μL) was used. Finally, the absorbance changes of MTT were detected at 490 nm in the Infinite 200 PRO microplate reader.

A fluorogenic substrate of CAS3 is Ac-DEVD-AMC, which is specifically cleaved by CAS3. A fluorogenic substrate of CAS9 is Ac-LEHD-AF, which is specifically cleaved by CAS9. After purchasing the Ac-LEHD-AF and Ac-DEVD-AMC fluorogenic substrates (Bachem AG, Bubendorf, Switzerland), they were added into 96-well black plates after adding the required reagents and cells, and their fluorescence analyses were performed in the Infinite 200 PRO microplate reader [37,38].

The mean values of MTT, apoptosis, and caspases are shown as % of control.

### 2.6. The Measurements of Mitochondria Membrane Potential (ΔΨm) and Cytosolic ROS (cROS) Levels

The increase in the JC-1 probe in the mitochondria indicates upregulation of ΔΨm [39,40]. The levels of ΔΨm were determined in cells after incubation with 2 μM JC-1 (Cayman Chemical, Inc., Istanbul, Turkey) for 30 min. After washing the JC-1 with 400 μL extracellular solution with Ca^2+^ (1.2 mM), the fluorescence intensities of JC-1 were recorded by the Infinite 200 PRO.

The generation of cROS was assayed in the Infinite 200 PRO using a dye (2′,7′- dichlorofluorescin diacetate, DCFH-DA) [39]. The nonfluorescent dye (DCFH-DA) is converted to fluorescent DCF by the oxidation products (ROS). The U937 cells were incubated in 96-well black plates with 4 μM DCFH-DA (30 min). After washing the cells with the extracellular solution, the fluorescence intensity of DCF was recorded by the Infinite 200 PRO [40].

After the calculation of fluorescence intensity/protein levels, the mean values of ΔΨm and cROS are shown as % of control.

### 2.7. The Analysis of Reduced Glutathione (rGSH), Glutathione Peroxidase (GSHPx), and Lipid Peroxidation (MDA)

The optic density changes of total protein (at 660 nm), MDA (at 532 nm), rGSH (at 412 nm), and GSHPx (at 412 nm) in the homogenate of U937 cells were spectrophotometrically analyzed (Cary 60 UV-Vis, Agilent, CA, USA). The details of analyses were given in previous studies [23,24,25]. The concentrations of rGSH and MDA are shown as the unit of μmol per g protein, while the enzymatic activity of GSHPx is shown as the international unit (IU) per g protein.

### 2.8. The Fluorescence Intensity Determination of cCa^2+^ Concentration

The acute INF-induced changes in cCa^2+^ concentration in U937 cells were measured in the LSCM800 using Fluo-3-AM dye [37,38]. After incubation of the cells with the dye (1 μM for 1 h) in the dark, the cells were washed with an extracellular solution, and then were excited in the LSCM800 using an argon laser (at 488 nm). After the stimulation of Ca^2+^ entry through the treatment of the TRPV1 agonist (CAP and 10 μM), the entry of Ca^2+^ was blocked by using the TRPV1 antagonist (CPZ and 0.1 mM). The green images of Fluo 3-AM were captured in the LSCM800 (at 515 nm) with an Axio Observer 7 microscope of Zeiss (Objective: 40 × 1.3 oil). The fluorescence intensities were measured in the captured image records using the Zeiss ZEN program (Jena, Germany). An arbitrary unit (a.u.) was used for the expression of the mean Fluo-3-AM result.

### 2.9. The Current Records of Electrophysiology (Patch-Clamp)

The whole cell records were saved in Patchmaster software by using an amplifier of HEKA EPC 10 (Lamprecht, Germany). During the current records of TRPV1, the voltage was clamped at –65 mV. The content details of the extracellular (patch chamber) and cytosolic (patch chamber) solutions were indicated in previous studies [23,24]. Before freezing the solutions, the pH of the solutions was adjusted to physiologic values (7.4 and 7.2). The osmolality levels of cytosolic and extracellular solutions were kept between 280 and 320 mOsmol using an osmometer (Osmomat 030, Gonotec GmbH, Berlin, Germany). CPZ (0.1 mM) was used for inhibiting the TRPV1 currents, but CAP (10 μM) was used for stimulating the channel. The results of TRPV1 current densities are shown in the unit pA/pF. For calculating the unit, the amplitude of maximal current (pA) in the U937 was divided by the capacitance of cells (pF).

### 2.10. The Analyses of Cytokines using ELISA

The supernatants of cell lysates were collected by ultrasonic homogenization (BANDELIN Electronic GmbH, Germany) and configuration (at 800 g for 5 min). The concentrations of TNFα (Cat #210-TA-100), IL1β (Cat #201-LB-025), and IL6 (Cat #PD6050) were analyzed in the ELISA program of Infinite 200 PRO at 450 nm using commercial kits (R&D Systems, MN, USA). After calculation of the levels of TNFα, IL1β, and IL6 from the standard curves, the unit as % of control was used for the expression of mean values.

### 2.11. Statistical Analysis

The obtained data are presented as the mean value ± standard deviation (SD). A one-way ANOVA test was used for detecting the presence of differences in the groups. The presence of an indicative statistical difference (*p* ≤ 0.05 value) was analyzed in the data using a nonparametric post hoc test (SPSS; Kruskal–Wallis).

## 3. Results

### 3.1. The Nontoxic Concentration of HCQ in the U937 Cell Line

**Figure 1:**
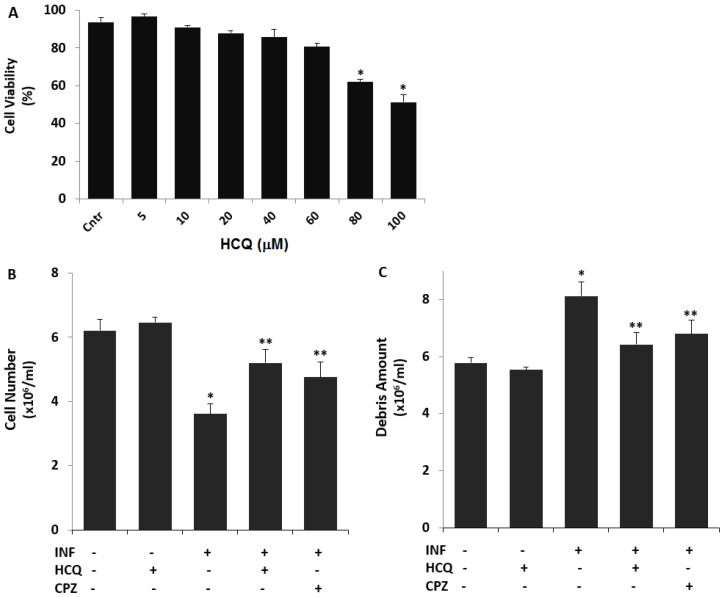
The non-toxic concentration of HCQ the U937 cell line. The effects of HCQ (60 μM for 48 h) and CPZ (100 μM for 30 min) on the cell number and debris amount in the U937 cell line treated with LPS (1 μM for 16 h). The non-toxic concentration of HCQ (**A**), cell number (**B**), and debris amount (**C**) were determined in the cells by using the CASY tone solution of CASY cell counter. (* *p* < 0.05 vs. Cntr. ** *p* < 0.05 vs. INF). The significance of *p*-values was analyzed by using the test of Kruskal–Wallis.

Nontoxic concentrations of HCQ were reported in different cell lines between 5 and 100 μM for 48 h [7,8,19]. As the first step of the current study, we investigated the nontoxic concentration of HCQ in the cells. The cells were treated with 5, 10, 20, 40, 60, 80, and 100 μM concentrations of HCQ for 48 h (Figure 1A). The cell viability levels were not significantly decreased in 60 μM HCQ as compared to the control group (*p* ≥ 0.05). When 80 or 100 μM HCQ was applied to the cells, the cell viability levels were significantly lower as compared to the control group (*p* ≤ 0.05). Hence, the cells were incubated in doses of 60 μM HCQ for 48 h.

### 3.2. The Acute INF-Induced Changes of Cell Number, Debris Amount, and Cell Volume Levels in the U937 Cells were Modulated by the Treatment of HCQ

The modulating role of HCQ in terms of the cell number and cell viability has been reported in several cell lines, but not U937 cells [19]. As the second step of the current study, we investigated acute INF-induced changes in cell number and debris amount by using the CASY tone count solution. The cell number (Figure 1B) was decreased in the group of acute INF as compared to the group of Cntr, although it was increased in the groups of acute INF + HCQ and INF + CPZ by the treatment of HCQ and CPZ (*p* ≤ 0.05). However, the amount of debris (Figure 1C) was increased in the group of acute INF, although its amount was decreased by the treatment of HCQ and CPZ (*p* ≤ 0.05).

### 3.3. Acute INF-Induced Expression Changes of Apoptotic Markers and TRPV1 in U937 were Modulated by the HCQ Treatment

**Figure 2:**
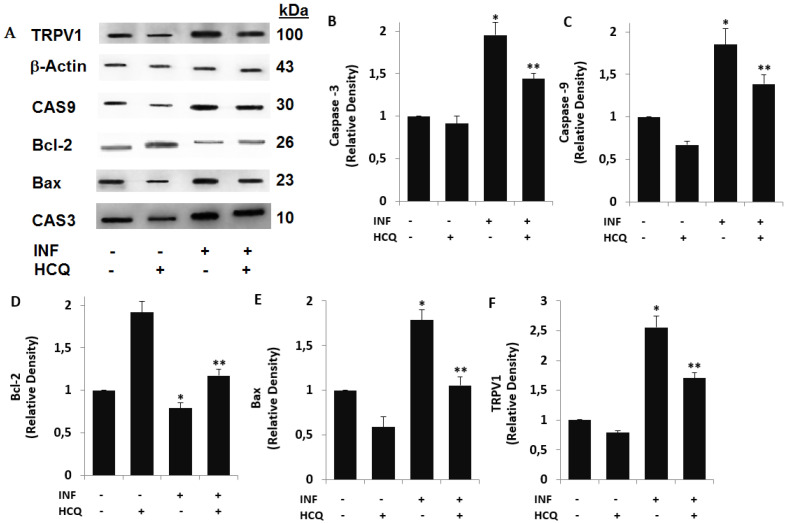
Acute inflammation (INF)-induced increases of TRPV1 and apoptotic factors were modulated by the HCQ treatment (60 μM). (Mean ± SD and *n* = 3). (**A**) The imaging total protein expression bands of Western blot. (**B**–**F**) indicate the mean expression concentrations of caspase -3 (CAS3), caspase -9 (CAS9), Bcl-2, Bax, and TRPV1 in the U937 monocytes, respectively. (* *p* < 0.05 vs. Cntr and HCQ. ** *p* < 0.05 vs. INF). The significance of *p*-values was analyzed by using the test of Kruskal–Wallis.

The accumulation data indicate that acute INF, via an increase in ΔΨm, induces a change in apoptosis markers (Bcl-2, Bax, CAS3, and CAS9) in several inflammatory cells [41,42]. The change in ΔΨm via the increase in acute INF-induced Ca^2+^ influx into the mitochondria is induced by the activation of several TRP channels, including the TRPV1 channel [43,44]. The protective action of HCQ against apoptosis via the modulation of Ca^2+^ influx was recently reported [45]. As to the acute INF-induced apoptotic pathways via the activation of TRPV1 channel, the involvement of HCQ has not been clarified yet. As a fourth step of the current study, we investigated the involvement of acute INF via the increase in TRPV1 expression in the apoptotic markers in the U937 monocytes. The protein band expressions of the apoptosis markers (Bax, Bcl-2, CAS3, and CAS9), control loading (β-actin)**,** and TRPV1 are shown in Figure 2A, although the row data of the bands are expressed in Figure S1. However, the band intensity values of CAS3, CAS9, Bcl-2, Bax, and TRPV1 expressions are indicated in Figure 2B–F, respectively. The expression levels of CAS3, CAS9, Bax, and TRPV1 were higher in the acute INF group compared with the control and HCQ groups, whereas their expressions were markedly (*p* ≤ 0.05) decreased in the cells by the treatment of HCQ. The expression level of Bcl-2 was lower in the acute INF group as compared to the control and HCQ groups, although the expression was increased in the group of INF + HCQ by the HCQ treatment (*p* ≤ 0.05).

### 3.4. The INF-Mediated Upregulation of Caspase, Apoptosis, JC1, cROS, and MDA Levels in the U- 937 Monocytes was Diminished via Ipregulation of rGSH and GSHPx by the HCQ and CPZ Treatments

**Figure 3:**
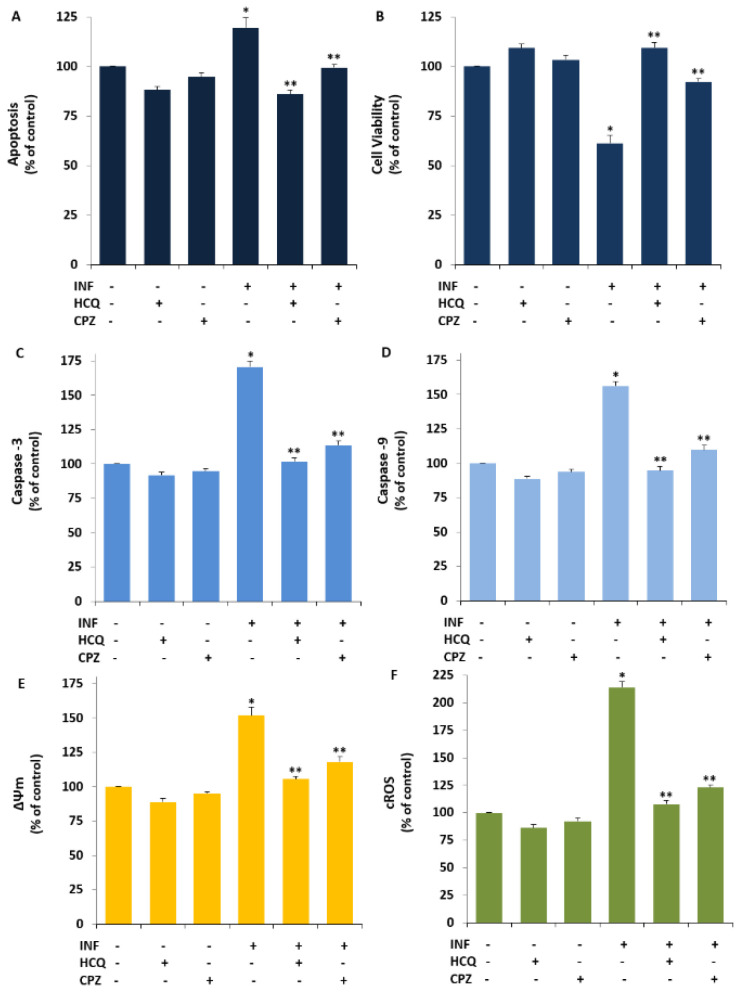
HCQ (60 μM) and CPZ (0.1 mM) attenuated acute INF-induced the increase of apoptosis level, caspase activates via the increases of cell viability in the U937 cell (Mean ± SD and *n* = 6). The column bars of (**A**–**F**) are indicating the changes of apoptosis, cell viability, CAS3, CAS9, ΔΨm, and cROS in the six groups. For the apoptosis analyses, a commercial kit (Biocolor APOPercentage) was used, although cell viability level was analyzed by using MTT. The Ac-DEVD-AMC and Ac-LEHD-AF fluorogenic substrates were used for the analyses of cleavage CAS3 and CAS9, respectively. For the determination of ΔΨm, and cROS, the stains of JC1 and DCFH-DA were used (* *p* ≤ 0.05 vs. Cntr, HCQ, and CPZ. ** *p* ≤ 0.05 vs. INF). The significance of *p*-values was analyzed by using the test of Kruskal–Wallis.

**Figure 4:**
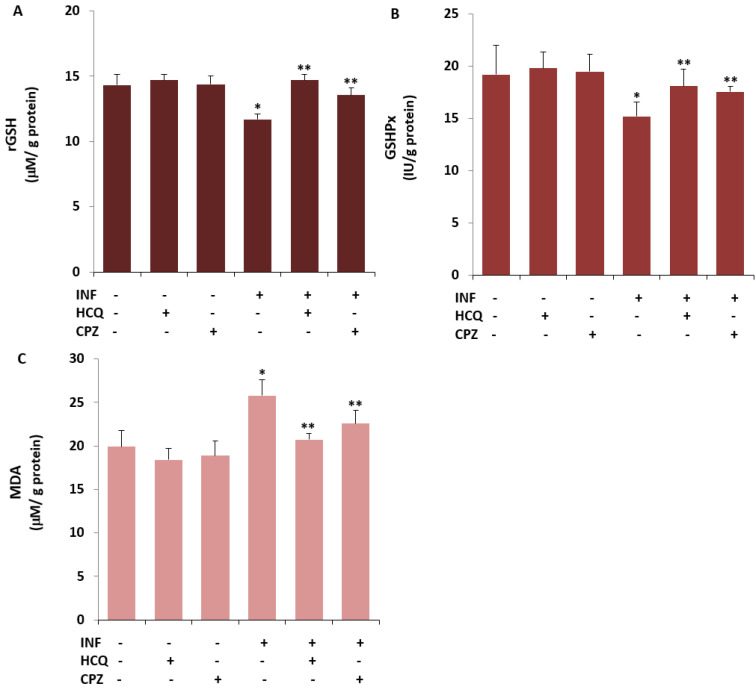
HCQ (60 μM) and CPZ (0.1 mM) attenuated INF-induced the increase of lipid peroxidation (MDA) in the U937 cell by the upregulation of reduced glutathione (rGSH) and glutathione peroxidase (GSHPx) (Mean ± SD and *n* = 6). The determinations of rGSH (**A**), GSHPx (**B**), and MDA (**C**) in the six groups were manually performed in the cells by using the spectrophotometer. (* *p* ≤ 0.05 vs. Cntr, HCQ, and CPZ. ** *p* ≤ 0.05 vs. INF).

Acute INF induces apoptosis and OS via the upregulation of caspases and TRPV1 activation but downregulation of rGSH and GSHPx in inflammatory cells and neurons [23,24,25,46,47]. However, there is no report of an effect of the TRPV1 channel blocker (CPZ) and HCQ on cell viability, CAP3, CAS9, apoptosis, ΔΨm, cROS, MDA, rGSH, and GSHPx in U937 monocytes. As the fifth step of the present study, we predicted the possible protective action of the HCQ and CPZ against INF-mediated apoptosis markers, OS values, ΔΨm, and glutathione redox systems via the inhibition of TRPV1 in the U937. The cell viability level was analyzed in the CASY cell counter by using the CASY tone solution. The concentrations of CAS3, CAS9, apoptosis, ΔΨm, and cROS were assayed in the cells using a microplate reader [23,24,40]. A spectrophotometer (Cary 60 UV- Vis) was used for the determination of rGSH, GSHPx, and MDA levels. In the present data, the levels of apoptosis (Figure 3A), CAS3 (Figure 3C), CAS9 (Figure 3D), ΔΨm (Figure 3E), cROS (Figure 3F), and MDA (Figure 4C) in the acute INF group were higher than those in the Cntr and HCQ groups (*p* ≤ 0.05), whereas the levels of cell viability (Figure 3B), rGSH (Figure 4A), and GSHPx (Figure 4B) were lower in the acute INF group than in the Cntr and HCQ groups (*p* ≤ 0.05). However, the upregulated values were decreased in the U937 monocytes by the HCQ and CPZ treatments. The concentrations of apoptosis, CAS3, CAS9, ΔΨm, cROS, and MDA were lower in the groups of HCQ and CPZ compared with the acute INF group, although the cell viability and concentrations of rGSH and GSHPx were higher in the groups of INF + HCQ or CPZ than in the INF group (*p* ≤ 0.05).

### 3.5. Acute INF-Mediated Increases in Ca^2+^ Fluorescence Intensity were Diminished via the Modulation of TRPV1 in U937 by HCQ and CPZ Treatments

**Figure 5:**
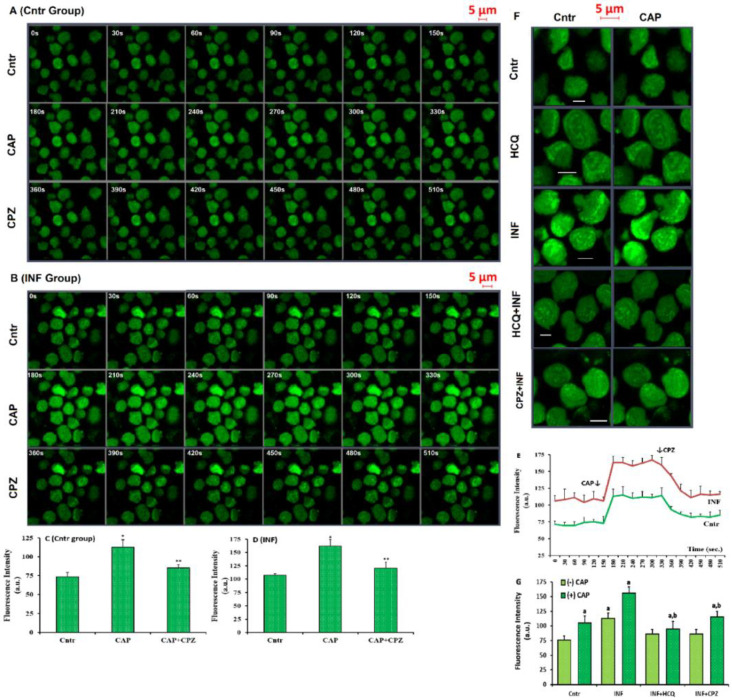
HCQ (60 μM for 16 h) modulated acute INF-caused increase of cCa^2+^ concentration in the U937 monocytes. (Mean ± SD). The cells were stained with Fluo 3-AM (1 μM for 1 h). The monocytes were stimulated by CAP (10 μM for 150s) and then, they were inhibited by CPZ (0.1 mM for 150s) in the confocal microscope (LSM800) with 40 × 1.3 oil objective. (**A**,**B**) Fluo 3-AM imaging and mean Ca^2+^ fluorescence intensity (arbitrary unit, a.u.) in the Cntr and INF groups, respectively. The capture time (second) of each image was indicated in the corner of each image by white numbers. (**C**,**D**) The mean concentrations of Ca^2+^ fluorescence intensities were indicated in the (**A**,**B**) by columns. (**E**) Fluo -3-AM images in the five groups (Cntr, HCQ, INF, HCQ + INF, and CPZ + INF). (**F**,**G**) The mean column graphics of Ca^2+^ fluorescence intensity in the Cntr and INF, respectively. The scale bar was kept as 5 µm. * *p* ≤ 0.05 vs. Cntr. ** *p* ≤ 0.05 vs. INF. **^a^** *p* ≤ 0.05 vs. CAP stimulation (-CAP). **^b^** *p* ≤ 0.05 vs. CAP stimulation (+CAP).

For the investigation of cCa^2+^ concentration, two Ca^2+^ influx analyses, namely Fluo -3-AM dye and patch-clamp electrophysiology, were performed in the cells. Before the analyses, the U937 cells were stained with Fluo-3-AM. The involvement of TRPV1 in the acute INF-induced cCa^2+^ concentration in the cells was tested using a LSM800 confocal microscope after its stimulation (via CAP) or inhibition (via CPZ). The images of Fluo-3-AM dye in the Cntr and acute INF are shown in Figure 5A,B respectively. The fluorescence intensity results in Cntr and acute INF were proven in the cells using columns (Figure 5C,D) and line (Figure 5E). The images of Fluo-3-AM in Cntr, HCQ, INF, HCQ + INF, and HCQ + CPZ were demonstrated in Figure 5F, whereas the mean values of fluorescence intensity in the groups were proven by columns (Figure 5G). The concentrations of fluorescence intensities were upregulated in the acute INF as compared with the Cntr and HCQ, and their concentrations were additionally upregulated in the acute INF by the agonist action of CAP (*p* ≤ 0.05). The upregulation of the fluorescence intensity was modulated in HCQ + INF and HCQ + CPZ by the HCQ and CPZ treatments, while they were lower in the HCQ + INF and HCQ + CPZ groups compared with acute INF only (*p* ≤ 0.05).

### 3.6. The Acute INF-Caused Upregulation of TRPV1 Current Densities was Downregulated in U937 Monocytes by HCQ Treatment

**Figure 6:**
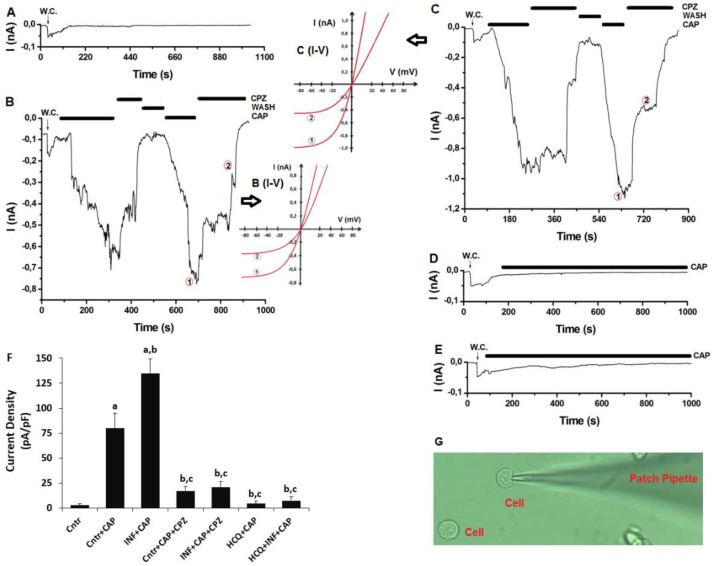
HCQ (60 μM for 48h) treatment modulated to the acute INF (LPS and 1 μg/mL for 16h)-caused upregulation of TRPV1 current densities in the U937 monocytes. (*n* = 3–6 and mean ± SD). The TRPV1 was activated by CAP (10 μM), although it was blocked by CPZ (100 μM). W.C.: whole cell. (**A**) Control (Cntr) (without CAP treatment). (**B**) Cntr + CAP group with the treatments of CAP and CPZ. (**C**) Acute INF + CAP group with the treatment of CAP and CPZ after the LPS treatment. (**D**). HCQ + CAP with CAP treatment after the HCQ incubation. (**E**) HCQ + acute INF + CAP group with the treatment of CAP and LPS after the HCQ treatment. (**F**) The mean densities of TRPV1 current. (**G**). Image of whole cell configuration. (^a^ *p* ≤ 0.05 vs. Cntr. ^b^ *p* ≤ 0.05 vs. the group of Cntr + CAP. ^c^ *p* ≤ 0.05 vs. INF+CAP).

In addition to the analyses of Fluo-3-AM, we further tested the Ca^2+^ influx in the U937 cells by using electrophysiology (patch clamp) analyses. In the absence of TRPV1 agonist (CAP), there was limited TRPV1 current density (up to 25 ± 12 pA) in U937 (Figure 6A). In the presence of CAP, there was TRPV1 activation in the cells between 0.82 and 1.85 min, and the densities of TRPV1 currents were higher in the Cntr + CAP group (79.82 pA/pF) compared with Cntr (4.25 pA/pF) (Figure 6A,B,F) (*p* ≤ 0.05). The densities of TRPV1 currents were additionally upregulated in acute INF + CAP (134.87 pA/pF) by the stimulation of CAP (Figure 6C (I–V)) (*p* ≤ 0.05). The densities of TRPV1 currents were lower in the Cntr + CAP + CPZ and acute INF + CAP + CPZ groups compared with Cntr + CAP and acute INF + CAP. In the presence of HCQ treatments, there was limited TRPV1 current via the CAP stimulation (Figure 6D–F). The mean densities of TRPV1 currents were lower in the HCQ + CAP (4.50 pA/pF) and HCQ + acute INF + CAP (7.33 pA/pF) groups compared with Cntr + CAP (79.82 pA/pF) and acute INF + CAP (*p* ≤ 0.05). The whole cell configuration is given in Figure 5G. The results for the patch clamp additionally demonstrated the modulator effect of HCQ on the acute INF-induced upregulation of Ca^2+^ influx via the stimulation of TRPV1 in the U937 monocytes.

### 3.7. Acute INF-Induced TRPV1 Stimulation Results in Cytokine Responses in U937 Monocytes: The Modulator Role of HCQ

**Figure 7:**
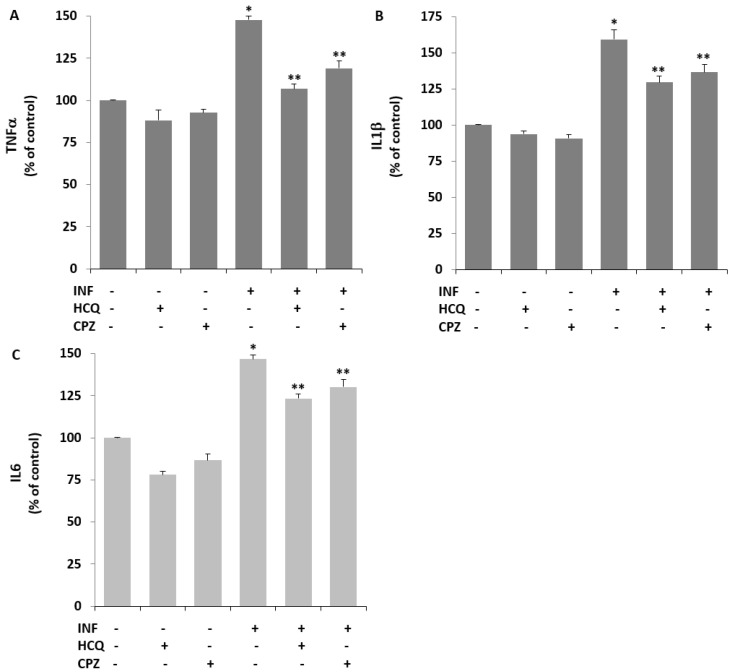
The acute INF-induced increases of cytokine generations were decreased in the U937 monocytes by the treatment of HCQ and CPZ. (Mean ± SD and *n* = 6). The generations of TNF-α (**A**), IL-1β (**B**), and IL-6 (**C**) were assayed in the Infinite PRO 200 by using the commercial ELISA kits. * *p* ≤ 0.05 vs. Cntr, HCQ, and CPZ. ** *p* ≤ 0.05 vs. INF).

The protective role of HCQ in the LPS-induced inflammatory cytokine response in several cell lines has recently been reported [17,18]. The protective action of HCQ on acute INF (LPS)-induced cytokine generation in monocytes, including U937 cells, has not yet been reported. To confirm this, the values of TNFα, IL1β, and IL6 were assayed by ELISA using commercial kits. Exposure of the cells to LPS, the values of inflammatory TNFα Figure 7A), IL1β Figure 7B), and IL6 Figure 7C) in the cells were upregulated in acute INF compared with control cells (*p* ≤ 0.05). In contrast, HCQ treatment again demonstrated a decrease in the values in U937 monocytes in the presence of LPS (*p* ≤ 0.05).

## 4. Discussion

Our data show that acute INF activates apoptotic, inflammatory, and oxidant action through acute INF-dependent excessive cROS, MDA, and cytokine generation, and Ca^2+^ influx in U937 human monocyte cells. Furthermore, we found that treatment inducing acute INF increased TRPV1 and apoptotic markers’ (CAS3, CAS9, Bax, and Bcl-2) concentrations via the downregulation of the rGSH level and GSHPx activity in U937 monocytes. However, the acute INF-induced OS cytotoxicity was diminished in U937 by treatment with HCQ and a TRPV1 inhibitor (CPZ). Finally, acute INF-caused activation of U937 human monocytes was induced by the stimulation of TRPV1. Treatment with HCQ and CPZ induced anti-inflammatory, anti-apoptotic, and antioxidant action via the inhibition of cROS, cytokines, caspases, and TRPV1. These results suggest that the acute INF-induced TRPV1 channel activation in U937 monocytes may lead to tissue death in inflammatory and excessive monocyte activation-induced diseases such as SLE, MS, and psoriasis.

Apoptosis is caused by different factors, including acute INF [48]. Apoptosis is mainly induced by extrinsic and internal molecular pathways. In the control of both pathways, the activation of caspases such as CAS3 and CAS9 plays an essential role [21]. In the intrinsic pathway of apoptosis, the increase in ΔΨm is via the accumulation of Ca^2+^ in the mitochondria. In turn, the increase in ΔΨm induces apoptosis via the upregulation of CAS3, CAS9, and Bcl-2 proteins. Of the members of the Bcl family proteins, Bcl-2 has anti-apoptotic properties, while Bax has apoptotic properties in several cells, including U937 cells [49]. In the present study, the expression concentrations of TRPV1 and apoptotic markers (CAS3, CAS9, and Bax) were upregulated by exposure to acute INF, but the expression level of the anti-apoptotic factor (Bcl-2) was downregulated. However, the expression of TRPV1, apoptotic, and anti-apoptotic markers was modulated in the cells after treatment with HCQ. Similar to the current results, the protective action of HCQ on the acute INF-induced apoptosis was reported in mice macrophages [50]. In the lymphocytes of patients with systemic SLE, the increase in apoptosis was diminished by the treatment with HCQ [14]. In renal carcinoma cells, the treatment with HCQ had different effects: apoptosis and the concentrations of, CAS3, CAS9, and Bax were upregulated by the HCQ treatment [51].

The TRPV1-blocking action of HCQ and CPZ in neurons and neuronal cells has been reported [26,52,53]. Excessive Ca^2+^ entry via the stimulation of TRPV1 has not only been demonstrated in several cell lines but has also been reported as participating in the induction of inflammation [23,24,30]. In the present study, the incubation of LPS for 24 h led to upregulated expression of TRPV1 and cytokines (TNFα, IL6, and IL1β), supporting the assumption that TRPV1 is involved in the LPS-caused acute INF of U937 macrophages. It has recently been indicated that CAP, via an increase in cytokine levels, activates TRPV1 in neurons and inflammatory cells [23,24,25], although conflicting results were also reported in U937 [26] and THP-1 malignant cell lines [35]. Here it was shown that CAP activated TRPV1 in the U937 cells. The modulating role of HCQ and TRPV1 inhibitor (CPZ) in cytokine generation in several cell lines has been reported [2,8,25,53,54]. Therefore, additional research was focused on the effect of TRPV1 antagonist (CPZ) and HCQ treatments on acute INF-induced cytokine generation in U937 cells. To determine whether the treatments for HCQ and TRPV1 channel inhibition also played a role in the anti-inflammatory activity in U937 cells, incubation with CPZ was used to treat U937 macrophages stimulated with acute INF. The anti-inflammatory action of LPS, assayed by the modulation of cytokine (TNFα, IL6, and IL1β) release in U-937 cells, was abolished in the cells by treatment with HCQ or CPZ. The data confirmed the anti-inflammatory effects of TRPV1 blockage (CPZ) and HCQ treatments detected in mice and human neutrophils, also indicating that the activation of the TRPV1 resulted in upregulated cytokine generation [2,8,15,25,53,54]. Thus, we have confirmed the role of TRPV1 in the anti- inflammatory action of HCQ in U937 monocyte signaling.

The excessive accumulation of cCa^2+^ in the mitochondria consequently induces excessive gating of the mitochondrial permeability transition pore via the downregulation of the antioxidant redox system [27]. In turn, this induces suppression of oxidative phosphorylation via an increase in the ΔΨm potential [11]. In several cells, the depolarization of ΔΨm induces increases in oxidant factors such as cROS, mROS, and MDA [2,23,24,25]. The upregulation of ΔΨm also participated in the LPS- caused OS cytotoxicity in the leucocytes [2,25]. Hence, the excessive generation of cROS and cytokines are two essential factors in the etiology of inflammatory and neurodegenerative diseases. Involvement of TRPV1 in acute INF-induced cROS and MDA production in the leucocytes and in mice was also reported [2,25,30,31]. However, the protective effect of HCQ has recently been reported to inhibit the OS via the regulation of the TRP channel and GSH system in renal tubular epithelial cells and human blood lymphocytes [55,56], although conflicting TRPV1 activator action for chloroquine was reported in the DRG of mice [57]. We, therefore, studied whether the modulating action of the HCQ in LPS-stimulated U937 monocytes was related to the downregulation of ΔΨm, cROS, and MDA via an increase in rGSH and GSHPx levels. Our data showed that the acute INF-induced cROS generation, ΔΨm, and MDA levels were decreased by the HCQ and CPZ treatments, while the concentrations of rGSH and GSHPx were reduced by the HCQ and CPZ treatments. Once again, treatment with HCQ and TRPV1 inhibitor (CPZ) resulted in a suppression of the acute INF-caused modulation of cROS generation, ΔΨm, and MDA levels via upregulation of rGSH and GSHPx.

## 5. Conclusions

We have demonstrated that the treatment of U937 monocytes with acute INF (LPS) increased the stimulation of TRPV1 and OS injury with increasing the cROS generation. The increase in apoptosis via the increase in caspase activation pathways resulted in monocyte death. The upregulation of cROS and MDA levels was associated with upregulated concentrations of TRPV1 expression and pro-apoptotic factors (CAS3, CAS9, and Bax) in response to the acute INF-caused overload of Ca^2^. More importantly, the treatment with HCQ and CPZ resulted in upregulated monocyte survival after treatment with LPS (see graphical abstract). Hence, TRPV1 was involved in the inflammation and OS injury of monocytes, which indicates that acute INF, via TRPV1 activation, may hasten monocyte activation in inflammatory diseases. Therefore, HCQ was able to inhibit acute INF-mediated TRPV1 stimulation and has potential to be used in the treatment of monocyte stimulation-caused inflammation and OS cell injury.

## Data Availability

The analyses and cell culture preparation were performed in the BSN Health, Analyses, Innovation, Consultancy, Organization, Agriculture and Industry, Ltd. (Isparta, Turkey). On reasonable request, the row data and details of analyses are available from M.G.

## Supplementary Materials

The following are available online at https://www.mdpi.com/article/10.3390/biology10100967/s1, Figure S1: The row data of TRPV1, caspase-3 (CAS -3), caspase -9 (CAS -9), Bcl-2, and Bax in the four groups of U937 human mono-cyte cells. (Mean ± SD and *n* = 3).

## Abbreviations

CAP	capsaicin
CAS3	caspase -3
CAS9	caspase -9
cCa^2+^	cytosolic free Ca^2+^
CPZ	capsazepine
cROS	cytosolic oxygen free radical species
HCQ	hydroxychloroquine
INF	inflammation
LPS	lipopolysaccharide
mROS	mitochondrial oxygen free radical products
MS	multiple sclerosis
NO	nitric oxide
OS	oxidative stress
SLE	lupus erythematosus
TRP	transient receptor potential
TRPV1	Transient receptor potential 1
ΔΨm	mitochondria membrane potential
