# Supplementary material for: Hydroxychloroquine Attenuates Acute Inflammation (LPS)-Induced Apoptosis via Inhibiting TRPV1 Channel/ROS Signaling Pathways in Human Monocytes
Source: Biology (Basel). 2021 Sep 27;10(10):967. doi: 10.3390/biology10100967 (PMC8533250; doi:10.3390/biology10100967)
Supplement: Supplementary file 1 [file biology-10-00967-s001.zip › biology-1366064-supplementary.pdf]

Hydroxychloroquine Attenuates Acute Inflammation (LPS)-Induced Apoptosis via Inhibiting TRPV1 channel/ROS Signaling Pathways in the human monocytes

Mustafa Güzel[1], Orhan Akpınar[2,3]

[1]Department of Medical Microbiology, Private Maltepe Medical Center, Istanbul, Turkey.
[2]Unit of Medical Microbiology, Department of Oral and Maxillofacial Surgery, Faculty of Dentistry, Suleyman Demirel University, Isparta, Turkey.
[3]Department of Medical Microbiology, Institute of Health Sciences, Suleyman Demirel University, Isparta, Turkey.

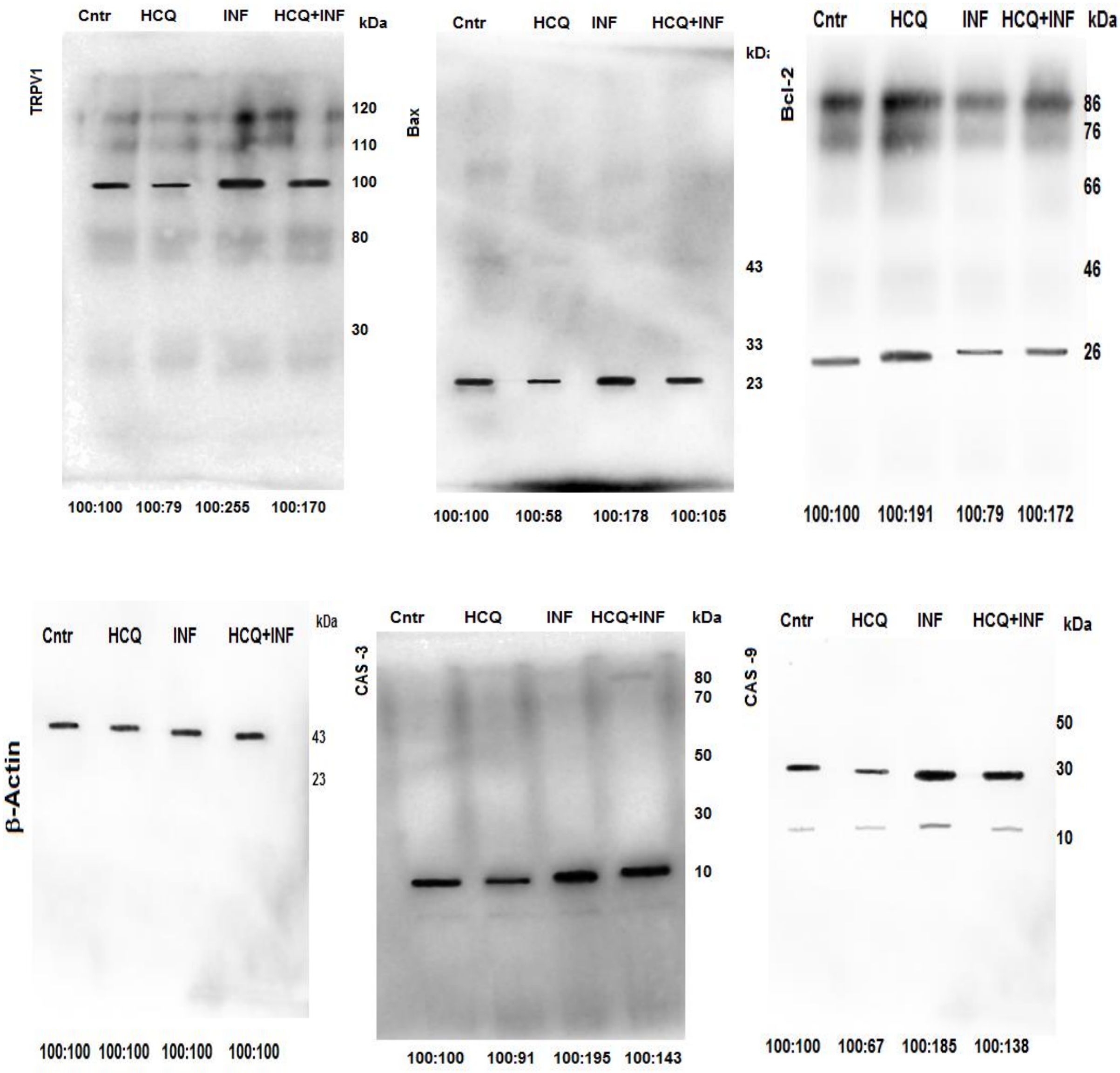

Supplementary Figure S1. The row data of TRPV1, caspase-3 (CAS -3), caspase -9 (CAS -9), Bcl-2, and Bax in the four groups of U937 human monocyte cells. (Mean ± SD and n=3). The standard western blot analysis was used for the expression of human protein levels in the cells of U937. The bands of β-actin protein were used as control. The dilution rates of the antibodies were kept between 1:200 and 1:1000. (b) The ratio of densitometry readings/ intensity ratio were measured by using the ImageJ software, and they were normalized to the β-Actin protein concentration. The unit of relative density changes (% of control) were used for the expression of the results.
